# Sialic Acid-Engineered IL4–10 Fusion Protein is Bioactive and Rapidly Cleared from the Circulation

**DOI:** 10.1007/s11095-019-2744-y

**Published:** 2019-12-26

**Authors:** Cristine Steen-Louws, Peter Boross, Judith Prado, Jan Meeldijk, Jurgen B. Langenhorst, Alwin D. R. Huitema, Marcel T. den Hartog, Louis Boon, Floris P. J. G. Lafeber, C. Erik Hack, Niels Eijkelkamp, Jelena Popov-Celeketic

**Affiliations:** 1 Center for Translational Immunology, UMC Utrecht, Utrecht University, Utrecht, Netherlands; 2 Department of Rheumatology and Clinical Immunology, UMC Utrecht, Utrecht University, Utrecht, Netherlands; 3 Department of Clinical Pharmacy, UMC Utrecht, Utrecht University, Utrecht, Netherlands; 4 Department of Pharmacy & Pharmacology, Netherlands Cancer Institute, Amsterdam, Netherlands; 5 Bioceros Holding B.V., Utrecht, Netherlands

**Keywords:** cytokines, inflammation, pharmacokinetics, sialylation, therapeutic protein

## Abstract

**Purpose:**

Modulating sialylation of therapeutic glycoproteins may be used to influence their clearance and systemic exposure. We studied the effect of low and high sialylated IL4–10 fusion protein (IL4–10 FP) on *in vitro* and *in vivo* bioactivity and evaluated the effect of differential sialylation on pharmacokinetic parameters.

**Methods:**

CHO cell lines producing low (IL4–10 FP lowSA) and high sialylated (IL4–10 FP highSA) fusion protein were generated. Bioactivity of the proteins was evaluated in an LPS-stimulated whole blood assay. Pharmacokinetics were studied in rats, analyzing plasma levels of IL4–10 FP upon intravenous injection. *In vivo* activity was assessed in an inflammatory pain mice model upon intrathecal injection.

**Results:**

IL4–10 FP lowSA and IL4–10 FP highSA had similar potency *in vitro*. The pharmacokinetics study showed a 4-fold higher initial systemic clearance of IL4–10 FP lowSA, whereas the calculated half-life of both IL4–10 FP lowSA and IL4–10 FP highSA was 20.7 min. Finally, both IL4–10 FP glycoforms inhibited persistent inflammatory pain in mice to the same extent.

**Conclusions:**

Differential sialylation of IL4–10 fusion protein does not affect the *in vitro* and *in vivo* activity, but clearly results in a difference in systemic exposure. The rapid systemic clearance of low sialylated IL4–10 FP could be a favorable characteristic to minimize systemic exposure after administration in a local compartment.

## Introduction

Interleukin-4 (IL-4) and IL-10, well-known anti-inflammatory cytokines, have potential for the treatment of inflammatory diseases. However, clinical studies with IL-4 or IL-10 have been disappointing (1–4). Several explanations for these results have been put forward including pharmacokinetic limitations, and lack of synergism with other cytokines when used as stand-alone drugs (5,6). IL4–10 FP, a fusion protein of IL-4 and IL-10, combines the effects of both cytokines in a single molecule (7,8), and overcomes some of the limitations of stand-alone IL-4 or IL-10.

Previously, we published the immunoregulatory activity of IL4–10 fusion protein *in vitro*. In whole blood and peripheral blood mononuclear cell (PBMC) cultures IL4–10 FP strongly inhibits the production of LPS-induced pro-inflammatory cytokines such as IL-1β, TNF, IL-6, and IL-8, while having minimal, if any, effect on the production of IL-1RA and sTNFR (8). In addition, the therapeutic potential of IL4–10 FP was demonstrated in several disease models both *ex vivo* and in vivo. The fusion protein inhibits pro-inflammatory cytokine production by rheumatoid arthritis (RA) and osteoarthritis (OA) synovial tissue and cartilage cultures (8,9). Systemic administration of IL4–10 FP attenuates experimental arthritis in mice (8). Intrathecal administration of IL4–10 FP remarkably reduces pain in various mouse models of inflammatory and neuropathic pain, as well as intra-articular administration in dogs with experimental OA (8,9). These findings suggest a potential of IL4–10 FP for the treatment of diseases, such as inflammatory pain and OA, where local application would be preferred over systemic administration.

Cytokines are potent modulators of immune and inflammatory reactions and other biological processes. Systemic administration of cytokines therefore may induce side effects resulting in severe morbidity or even death (10–12). Although systemic administration of IL-4 and IL-10 was well tolerated (1,4), systemic exposure to IL4–10 FP upon leakage of the local compartment may result in undesirable side effects, such as an increased risk of developing allergy. Therefore, we evaluated the feasibility to generate a modified IL4–10 FP that is active upon local administration but rapidly cleared from the circulation upon entering the bloodstream. IL4–10 FP has three potential N-linked glycosylation sites that can be occupied with complex glycans, which may be capped with a terminal sialic acid. Glycoproteins with glycans insufficiently capped with sialic acids are rapidly removed from the circulation by asialoglycoprotein receptors (ASGPR) in the liver (13). Thus, engineering of sialylation can be used to modulate the systemic exposure of a glycoprotein. To what extent sialylation impacts the bioactivity of IL4–10 FP is, however, not known. We hypothesized that selection of cell lines that produce IL4–10 FP with glycans insufficiently capped by sialic acids, may yield a glycoform of IL4–10 FP that is bioactive upon local administration, but is rapidly removed from the circulation when leaking from the local compartment. To test this hypothesis, we generated Chinese hamster ovary (CHO) cell lines producing IL4–10 FP. CHO cells provide a popular mammalian host for large-scale commercial production of therapeutic proteins as these cells are safe and allow high volumetric yields (14,15). We generated CHO cells lines producing high- and low-sialylated IL4–10 FP (IL4–10 FP highSA and IL4–10 FP lowSA, respectively). Recombinant IL4–10 FP glycoforms were tested for functional activity *in vitro*, for pharmacokinetics in a rat model, and for efficacy upon intrathecal injection in a mouse model of persistent inflammatory pain.

## Methods

### Generation of CHO Cell Lines

CHO^BC^® cell lines producing IL4–10 FP were generated in the facilities of Bioceros, Utrecht, the Netherlands. Unless indicated otherwise, CHO^BC^® cells, thawed from a working cell bank, were cultured and expanded in serum-free ProCHO™-5 medium (Lonza) supplemented with 4 mM L-glutamine (Gibco) with pluronic F-68 (Life Technologies), at 0.1 or 0.2%, *w*/*v* (culture medium) in a humidified CO_2_ incubator. Cell number and viability were assessed with a CASY counter (Roche Innovatis AG). Levels of IL4–10 FP produced by the cells were measured in the supernatant with a human IL-10 ELISA kit (Sanquin, Cat# M9310) according to manufacturer’s instructions.

To generate IL4–10 FP producing cell lines, CHO^BC^® cells were transfected with a proprietary expression plasmid containing cDNA encoding IL4–10 FP, and with a plasmid with cDNA encoding α2,3-sialyltransferase (ST3GAL_IV) using an AMAXA Nucleofector II device (Lonza). After transfection, cells were pooled and seeded at 1.0 × 10^6^ viable cells (vc) per ml culture medium in T-flasks. Cells were incubated for 48 h and seeded in 96-well plates at 300 to 3000 vc/well in culture medium supplemented with Zeocin (Invitrogen) and Blasticidin (Invitrogen) (selection medium). The cells were incubated for ~3 weeks. Visible cell clones were then transferred into 24-well plates containing 2 ml fresh selection medium and screened for IL4–10 FP levels (IL-10 ELISA). Best producing clones were selected, cultured in 6-well plates and expanded into 25 ml T-flasks (4-day culture). Subsequently, they were adapted to culture under shaking conditions and cultured for 7 days while cell density, viability and production were monitored at various intervals (7-day culture). In addition, sialylation of IL4–10 FP produced by different clones was analyzed using lectin-based ELISAs (see below). Finally, two cell lines producing IL4–10 FP with respectively low and high sialylation were selected.

### Glycan Analysis with Lectin-Based ELISAs

Sialylation of IL4–10 FP produced by the CHO^BC^® clones was evaluated using ELISA-type assays with the galactose-binding *Erythrina Cristagalli* Lectin (ECL), or the α2,3 sialic acid binding *Maackia amurensis* Lectin II (MAA). CHO^BC^® cell supernatant to be tested was diluted in PBS to yield a concentration of 10 μg/ml IL4–10 FP. 100 μl of diluted supernatant was coated in duplicate in the wells of maxisorp plates (Nunc). After O/N incubation at 4°C, plates were washed 3 times with PBS containing 0.05%, *w*/*v*, Tween-20 (Merck), followed by incubation with 200 μl/well PBS 0.1%, *w/v*, Tween-20 (PBS-T), 1 h at room temperature (RT), while gently shaking (300 rpm). 100 μl of biotinylated ECL or MAA (Vector Laboratories), diluted 1 to 10.000 and 1 to 3000 in PBS-T, respectively, were added to the plates, which were subsequently incubated for 1 h at RT. Bound biotinylated lectins were detected by incubation with Streptavidin poly-HRP (1:10,000, Sanquin) in PBS-T for 30 min at RT, followed by incubation with TMB (Invitrogen). The reaction was stopped with 1 M H_2_SO_4_ and the absorbance at 450 nm was measured. The ratio MAA/ECL, calculated using the OD450 values of bound MAA and bound ECL, is indicative for the sialylation ratio of the IL4–10 fusion protein.

### Purification of Recombinant IL4–10 FP

IL4–10 FP was purified from conditioned medium using affinity chromatography according to a previously published protocol (16). In short, protein G-purified, in-house made mouse anti-IL4 mAb was coupled to CNBr-activated Sepharose 4B (GE Healthcare Life sciences), according to manufacturer’s instructions, and packed into a column. To prevent non-specific binding of proteins the column was flushed with phosphate buffered saline (PBS, pH 7.4), containing 1%, *w*/*v*, Bovine Serum Albumin (BSA), and then equilibrated with PBS without BSA. Concentrated conditioned medium containing IL4–10 FP was loaded on the column, which was then washed with PBS. Bound IL4–10 FP was eluted with 0.1 M Glycine, pH 2.25. Fractions were immediately neutralized with 1 M Tris, pH 9.0 and dialyzed in PBS. Fractions containing IL4–10 FP were pooled, assessed for purity and protein content by running a protein gel (see below), a BCA protein assay (Thermo Scientific) and the IL-10 ELISA, aliquoted and stored at −80°C until further analysis.

### Protein Electrophoresis, Protein Stain & Western Blot

Cell supernatants were diluted 1 to 1.33 in Laemmli sample buffer (BioRad), containing 100 mM Dithiotreitol (Sigma-Aldrich), incubated for 10 min at 100°C, whereafter 10 ul was loaded on a 12% polyacrylamide Gel (Mini-PROTEAN-TGX, BioRad). After electrophoresis, the gel was either stained with Instant Blue (Expedeon) for visualization of total protein content, or prepared for Western blotting in order to specifically detect IL4–10 FP. Blotting was performed using the Trans-Blot system (BioRad) in combination with a 0.2 μm nitrocellulose-membrane Transfer Pack (BioRad), according to manufacturer’s instructions. After transfer, the membranes were blocked in 4% milk (Elk, Campina) in PBS 0.1%, *w*/*v*, Tween-20 (PBS-T). The membrane was incubated with the primary Ab mouse anti-human IL-4 (1:400, Santa Cruz Biotechnology, Clone 13Z07) or mouse anti-human IL-10 (1:100, Santa Cruz, Clone 3C12C12), in 1% milk in PBS-T, followed by HRP-conjugated goat anti-mouse IgG (1:2000, Santa Cruz). To visualize the bands ECL Western blotting substrate was added according to manufacturer’s protocol (Pierce, Thermo Scientific). Stained protein gels and Western blots were imaged using the ChemiDoc MP system (BioRad).

### Functional Activity of IL4–10 FP

Heparinized human blood obtained from healthy volunteers was diluted 1:10 in RPMI1640 medium (Invitrogen), supplemented with 1% penicillin/streptomycin (P/S, PAA Laboratories) in 48-well culture plates (Nunc). Lipopolysaccharide (LPS, Sigma-Aldrich) was added at 10 ng/ml, as well as low and high sialylated IL4–10 fusion protein at 0.01–3 nM (0.3–270 ng/ml), final concentrations. As control, HEK293 produced IL4–10 fusion protein was taken along (16). After 18 h incubation at 37°C, 5% CO_2_, culture supernatant was collected and tested for TNF concentration with ELISA (DiaClone, Cat# 851570020). The percentage inhibition was calculated relative to TNF production by LPS in absence of IL4–10 FP.

### Deglycosylation of IL4–10 FP

IL4–10 FP was enzymatically deglycosylated using PNGaseF (New England BioLabs) according to manufacturer’s protocol. Effectiveness of deglycosylation was monitored by Western blotting.

### Rat Model to Evaluate the Pharmacokinetics of IL4–10 FP

Clearance of IL4–10 FP from the circulation was studied in Wistar Crl:WI female rats (mean body weight 205 ± 15 g; Charles River Laboratories). The study was approved by the animal ethical committee (project number AVD11500201744) and performed at the animal facility of the Utrecht University. Rats were acclimatized for 1 week prior to the experiment. Rats were intravenously injected via the tail vein with 5 μg IL4–10 FP lowSA or IL4–10 FP highSA, or with a mixture of 5 μg IL-4 (eBioScience) and 5 μg IL-10 (Invitrogen) in a volume of 200 μl PBS. Some rats were injected intravenously with 30 mg asialofetuin (Sigma-Aldrich) in 0.5 ml PBS 10 min prior to injection of low sialylated IL4–10 FP, to saturate the ASGPR. Blood was collected from the tail using a winged infusion system (150 ul per timepoint) in lithium-heparin tubes (Sarstedt) either at 5, 15, 30 and 120 min, or 10, 20, 60 and 120 min after injection of IL4–10 FP and plasma was prepared by centrifugation for 5 min at 2000 g. IL4–10 FP and IL-10 levels were measured with the IL-10 ELISA. IL-4 levels were measured with a human IL-4 ELISA (Sanquin, Cat# M9314) according to manufacturer’s instructions. Pharmacokinetics were evaluated using non-linear mixed effects modeling (with NONMEM v.7.3). Models were parameterized in terms of fixed effects (population mean) and two levels of random effects; per subject (inter-individual variability [IIV]) and per measurement (residual variability). A one-compartmental model with linear elimination was used as a structural model. Herein, clearance was assumed to be different for IL-10, IL-4, and IL4–10 FP. For IL4–10 FP, the clearance was further differentiated into a slow and fast route, where the fast route represented rapid ASGPR-mediated clearance. The fraction of the dose administered that eliminated via the fast route was estimated separately for high and low sialylated IL4–10 FP. For the rats that received asialofetuin prior to injection with IL4–10 FP, the proportion fast clearance was assumed to be zero. The 95% confidence interval of parameter estimates were calculated by sampling-importance-resampling (SIR). Half-life was calculated using the formula: T_1/2_ = 0.693*(V/CL), where V is the distribution volume (ml) and CL is the clearance rate (ml/min).

### Mouse Model for Inflammatory Pain

The efficacy of IL4–10 FP to reduce persistent inflammatory pain was tested in the carrageenan-induced inflammatory pain model as described before (7). Briefly, carrageenan (2%, 20 μL; Sigma-Aldrich) was injected in both hind paws of 8–12 weeks old C57Bl/6 mice to induce persistent inflammatory pain. Six days after carrageenan injection, 1 μg low or high sialylated IL4–10 FP (in 5 μl PBS; *n* = 9 for both groups) or vehicle (saline; *n* = 7) was injected intrathecally. Mechanical hypersensitivity was measured using von Frey hairs (Stoelting), and the 50% paw withdrawal threshold was calculated using the up-and-down method (17). Investigators performing the behavioral assays or assessing outcome were blinded for treatment. Experiment was performed in two sessions and results (group mean) were analyzed with a repeated measures 2-way ANOVA with the Geisser-Greenhouse correction, followed by a Tukey’s post hoc test, with individual variances computed for each comparison.

## Results

### CHO Cell Lines Producing IL4–10 FP

**Fig. 1:**
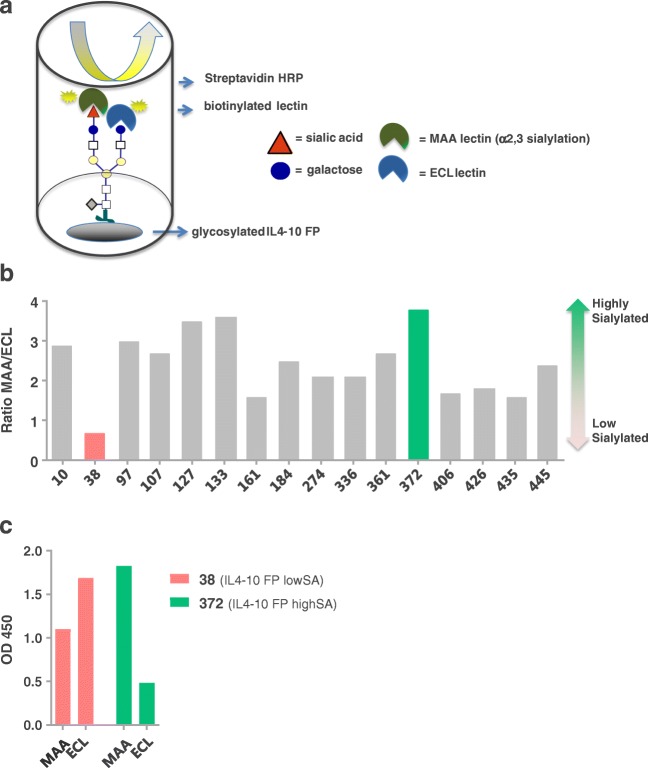
**Differential sialylation of IL4–10 FP produced by various CHO cell lines.** IL4–10 FP was produced by various CHO cell lines, and evaluated for sialylation using lectin-based analysis. Conditioned medium containing IL4–10 FP was coated on ELISA plates, biotinylated MAA-lectin and biotinylated ECL-lectin were used to detect end-standing sialic acids or end-standing galactose residues, respectively (**a**). The ratio of 450 nm optical densities between MAA and ECL reflects the degree of sialylation of the cell lines (**b**). IL4–10 FP produced by the cell lines CHO38 and CHO372 has least (IL4–10 FP lowSA) and most (IL4–10 FP highSA) end-standing sialic acids, respectively. The raw OD450 values show that IL4–10 FP lowSA contains some glycans capped with sialic acids, while IL4–10 FP highSA still contains some end-standing galactose residues (**c**).

CHO cell lines producing IL4–10 FP were generated according to the described procedures. During screening for production and sialylation, lead cell lines were selected using a combination of IL-10 and lectin-based ELISAs. The sialylation of recombinant IL4–10 FP, produced by 16 finally selected cell lines is shown in Fig. 1b. In the lectin-based ELISA, bound recombinant protein is fixed to an ELISA plate and assessed for sialylation using MAA and ECL lectins, which are specific for end-standing α2,3-sialic acid or galactose, respectively. The ratio of the OD450 values obtained with both lectins, was taken as a measure for sialylation of IL4–10 FP (Fig. 1a). Moderate sialylation, with a MAA/ECL ratio of 2, was observed for 8 of the clones. One clone, CHO38, had a much lower MAA/ECL ratio (0.7), indicating that this cell line produced low sialylated IL4–10 FP (IL4–10 FP lowSA), while a relative high MAA/ECL ratio (3.8) was observed for clone CHO372, indicative for the production of high sialylated IL4–10 FP (IL4–10 FP highSA). Indeed, in case of clone CHO38, preferential ECL binding suggested the presence of IL4–10 FP with glycans containing end-standing galactose, produced by this cell line. Conversely, IL4–10 FP produced by the cell line CHO372 had most glycans capped with α2,3 sialic acid, as shown by the high MAA-lectin OD450 (Fig. 1c).

**Fig. 2:**
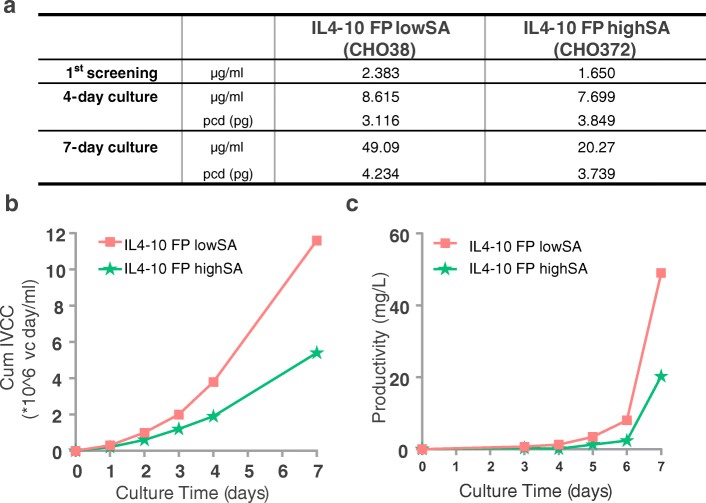
**Viability and productivity of CHO cell lines 38 and 372.** Productivity of the cell lines CHO38 and CHO372 producing respectively IL4–10 FP lowSA and IL4–10 FP highSA, during cell-line development (**a**). Cumulative integral viable cell concentration (**b**) and volumetric yield during 7-days culture are plotted (**C**). Production of IL4–10 FP was measured using a human IL-10 sandwich ELISA.

Assessment of the productivity during cell line generation indicated specific IL4–10 FP production by cell line CHO38 of 3.1 pg/cell/day (pcd) as a result of the 4-day culture and 4.2 pcd during the 7-day culture. For CHO372, the specific IL4–10 FP productivity was 3.9 and 3.7 pcd during 4-day culture and 7-day culture, respectively (Fig. 2a). These results demonstrate that IL4–10 FP production during different cultures and conditions is consistent and sufficiently high for further development. Cumulative growth curve and volumetric productivity over time, during the 7-days culture of both cell lines are presented in Fig. 2b and c and confirm, together with the data in Fig. 2a that cell viability and productivity of the selected clones were as expected.

### Characterization of IL4–10 FP Produced by CHO38 and CHO372 Cell Lines

**Fig. 3:**
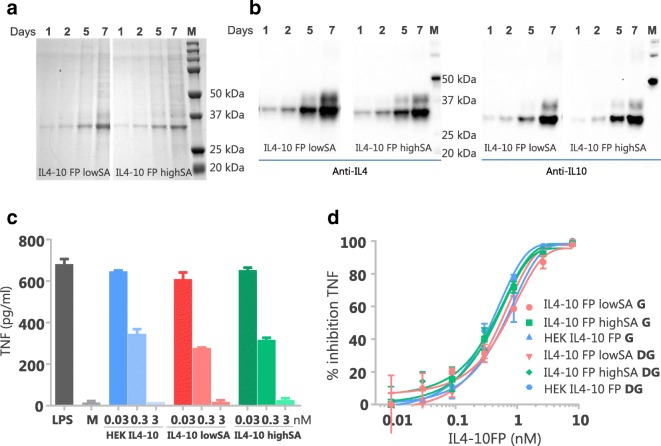
**Characterization of IL4–10 FP produced by the cell lines CHO38 and CHO372.** Production of IL4–10 FP during 7 days culture by CHO38 (IL4–10 FP lowSA) and CHO372 (IL4–10 FP highSA), as shown on protein-stained SDS-PAGE (**a**; IL4–10 FP migrates at ~33kD) and immunoblot with anti-IL4 (B; left) and anti-IL10 (**b**; right). IL4–10 FP was purified from culture medium of each cell line using affinity chromatography with anti-IL4 mAb, and incubated overnight in whole blood together with LPS, as was purified IL4–10 FP produced by HEK293 cells. Cultures were measured for TNF production. The activity of IL4–10 FP is shown by means of TNF inhibition compared to production with LPS only (**c**). The effect of deglycosylation (“DG”) of IL4–10 FP on the functional activity in the whole blood assay was tested upon PNGaseF treatment of IL4–10 FP highSA, IL4–10 FP lowSA and HEK293 produced IL4–10 FP. As controls, glycosylated (“G”) IL4–10 FP incubated under equal conditions but without PNGaseF, was also tested (**d**). Data in C and D represent mean and SD of duplicates.

Both IL4–10 FP lowSA and IL4–10 FP highSA appeared as protein bands with an apparent mass of ~34 kDa on a protein-stained SDS PAGE gel (Fig. 3a) as well as on Western blot, where both recombinant proteins were identified with an anti-IL4 and anti-IL10 antibody (Fig. 3b**)**. Purification of IL4–10 FP lowSA and IL4–10 FP highSA from culture medium using affinity chromatography via an anti-IL4 antibody recovered ~50% of the recombinant protein and yielded ~90–95% purity (data not shown). Both IL4–10 FPs inhibited LPS-induced TNF production in whole blood in a concentration dependent manner, with maximal inhibition at 3 nM (Fig. 3c**)**. The inhibitory activity was comparable between IL4–10 FP lowSA and IL4–10 FP highSA. Furthermore, both glycoforms inhibited LPS-induced TNF production similar to that of HEK293-produced IL4–10 FP (used in our previous *in vitro* and *in vivo* studies) (16). These data indicate that the cellular platform (HEK293 or CHO cells) used for production, nor differential sialylation affect the potency of IL4–10 FP. Next, we further evaluated whether glycosylation affects the functional ability of IL4–10 FP by deglycosylation of IL4–10 FP produced by both clones with PNGaseF. Importantly, deglycosylation did not affect the dose-dependent inhibition of LPS-induced TNF production by both IL4–10 FP lowSA and IL4–10 FP highSA. (Fig. 3d). These results indicate that neither glycosylation nor sialylation affect the inhibitory capacity of human IL4–10 FP *in vitro*.

### Clearance of IL4–10 FP Is Dependent on Sialylation

In a pharmacokinetics study we investigated whether the differential sialylation of IL4–10 FP affects its clearance from the circulation. Each IL4–10 FP preparation was injected in rats intravenously (5 μg via the tail vein), and the circulating concentration of IL4–10 FP in serial plasma samples was assessed by ELISA.

**Fig. 4:**
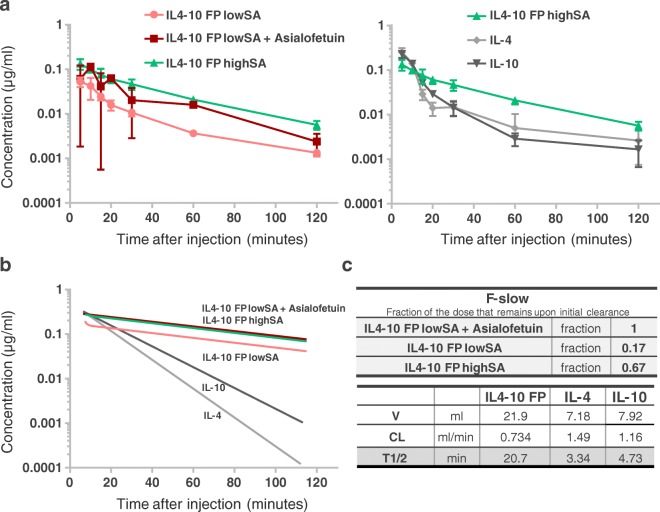
**Clearance of high and low sialylated IL4–10 FP upon intravenous injection in rats.** Wistar rats were injected with IL4–10 FP highSA or IL4–10 FP lowSA (20 μg each) or with a combination of IL-4 and IL-10 (10 μg each) via the tail vein. Some rats injected with IL4–10 FP lowSA were pretreated with asialofetuin, to block ASGPR in the liver. Levels of IL4–10 FP, IL-4 and IL-10, were then measured in plasma samples collected at various time intervals (**a**). Curves represent mean and SD of 6 animals in total, composed of samples taken at alternating time-points (3 animals per time-point). The alternating sampling gives some variation in the IL4–10 FP lowSA group, pre-injected with Asialofetuin. Pharmacokinetic parameters were calculated using a one-compartmental model with linear elimination in NONMEM (**b**, **c**).

Concentrations of IL4–10 FP showed an initial rapid decrease upon administration of IL4–10 FP lowSA presumably reflecting fast hepatic clearance (Fig. 4a and b). Administration of asialofetuin in animals prior to injection with IL4–10 FP lowSA largely prevented the fast elimination of IL4–10 FP, indicating that the rapid clearance of IL4–10 FP lowSA in part is mediated by the ASGPR. However, a small fraction of the administered dose shows a slow clearance, similar to the clearance of IL4–10 FP lowSA in combination with asialofetuin (Fig. 4c; estimated fraction “F-slow” is 0.17, 95% confidence interval 0.14–0.22). This indicates that the elimination route of that fraction of IL4–10 FP lowSA is not sialylation-dependent, which can be explained by the presence of a small fraction of sialylated IL4–10 FP in the batch of IL4–10 FP lowSA. The slow elimination pattern was also observed for IL4–10 FP highSA. However, the fraction of highly sialylated IL4–10 FP in this product was much higher, as expected (Fig. 4c; estimated fraction “F-slow” is 0.67, 95% confidence interval 0.5–0.86). The slow elimination of IL4–10 FP (most likely representing the elimination of the highSA fraction of both products) resulted in a half-life of 20.7 min (Fig. 4b and c). In contrast, half-lives of wild-type IL-10 and IL-4 were much shorter and were estimated at 4.73 min and 3.34 min, respectively (Fig. 4b and c).

### IL4–10 Inhibits Persistent Inflammatory Pain in Mice

**Fig 5:**
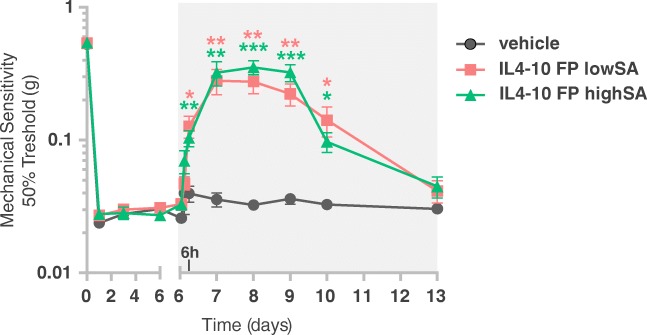
**Differential sialylation does not affect the efficacy of intrathecally injected IL4–10 FP to inhibit established persistent inflammatory pain in mice.** Persistent inflammatory pain was induced by an intraplantar injection of 20 μl of 2% carrageenan. Six days (d) after carrageenan injection, mice received an intrathecal injection of 1 μg IL4–10 FP highSA (*n* = 9), IL4–10 FP lowSA (n = 9), or vehicle (*n* = 7). Mechanical hypersensitivity was measured over time using the von Frey test. Results were expressed as 50% threshold (g) and represent mean and standard error of two combined experiments. Colored asterisks mark statistical differences compared to vehicle-treated mice. * = *p* ≤ 0.05; ** = *p* ≤ 0.01; *** = *p* ≤ 0.001.

Next, we tested whether low or high sialylation of IL4–10 FP affects its therapeutic potential to suppress inflammatory pain *in vivo*. Intrathecal injection of IL4–10 FP produced in HEK293 cells reduces pain in various mouse models, including models for persistent inflammatory pain (7). For the current study we evaluated the efficacy of CHO produced IL4–10 FP lowSA and IL4–10 FP highSA to reduce carrageenan-induced inflammatory pain. Intraplantar injection of carrageenan increased sensitivity to mechanical stimuli in mice, indicating development of mechanical hyperalgesia. Intrathecal injection of IL4–10 FP at day 6 after carrageenan injection inhibited established mechanical hyperalgesia for 3–4 days. Importantly, IL4–10 FP highSA and IL4–10 FP lowSA suppressed persistent inflammatory hyperalgesia to the same extent and duration (Fig. 5). These results indicate that the efficacy of IL4–10 FP upon intrathecal injection is not affected by sialylation in this inflammatory pain model.

## Discussion

Intrathecal administration of IL4–10 FP inhibits hyperalgesia and allodynia in mouse models of chronic pain in a superior fashion compared to stand-alone wild-type IL-4 or IL-10, or a combination therapy of both cytokines (7). For clinical application of IL4–10 FP to treat pain by local therapy it might be important to limit systemic exposure to avoid potential side effects. Here we describe the development of two IL4–10 FP glycoforms expressed by CHO, one with glycans poorly capped with sialic acids (IL4–10 FP lowSA) and one with glycans that are mainly capped with sialic acids (IL4–10 FP highSA). The poorly sialylated IL4–10 FP lowSA had a faster clearance from the circulation compared to the highly sialylated IL4–10 FP highSA, whilst retaining its functional ability to inhibit inflammatory responses *in vitro* and to inhibit persistent inflammatory pain *in vivo*.

Depending on culture conditions, expression of therapeutic glycoproteins in CHO and other mammalian cell lines often yields proteins with incomplete capping of glycans by sialic acids (18). Incomplete sialylation of their glycan residues may lead to the rapid removal of therapeutic proteins from the circulation through the uptake by the asialo-glycoprotein receptor (ASGPR) in the liver (19,20). This mechanism may account for a serum half-life of only a few minutes. As biologics are nowadays mostly administered systemically, improving sialylation in order to increase systemic exposure is a well-known tool that is applied to improve the pharmacokinetic characteristics of therapeutic proteins. On the other hand, if the treatment with therapeutic proteins needs to be local, an under-sialylated form of a therapeutic protein would be a drug of choice. To evaluate the effect of differential sialylation of IL4–10 FP, we engineered the CHO cells by introducing co-transfection with α2,3-sialyltransferase. Most proteins obtained from cell lines using this strategy were indeed significantly sialylated. Cell line CHO372 showed most extensive sialylation of IL4–10 FP and this cell line was selected to produce a research batch of IL4–10 FP highSA. However, co-transfection of cells with α2,3 sialyltransferase does not guarantee cell lines that produce high-sialylated glycoprotein. Rather, this strategy may result in a variable number of cell lines with poor sialylation capacity (28). We indeed identified a cell line, CHO38, that produced IL4–10 FP which predominantly bound ECL lectin, suggesting the presence of glycans with mainly end-standing galactose. This cell line was used to produce a research batch of IL4–10 FP lowSA.

Although glycosylation of proteins affects their systemic exposure *in vivo*, the different capping of glycans did not affect the anti-inflammatory properties of IL4–10 FP *in vitro* and *in vivo*. In the whole blood assay, the potency of both glycoforms of IL4–10 FP appeared to be similar. This is in line with the findings that wild-type IL-4 and IL-10, expressed in *E.coli* and therefore non glycosylated, are functionally active (21,22). Furthermore, both IL4–10 FP lowSA and IL4–10 FP highSA were equally able to inhibit chronic inflammatory pain in mice.

The contribution of differential glycosylation to the systemic exposure was shown in the pharmacokinetics study by the rapid initial elimination of IL4–10 FP lowSA (upon injection, only 17% was left in circulation). Blockade of the ASGPR with asialofetuin (23) in rats that were injected with IL4–10 FP lowSA, yielded a similar clearance curve as compared to IL4–10 FP highSA, indicating that the differences in initial clearance between both glycoforms were mostly due to interaction of IL4–10 FP lowSA with the ASGPR in the liver. For IL4–10 FP highSA, the initial elimination was much lower, resulting in 67% IL4–10 FP in circulation upon injection. The fraction of the dose that remained in circulation was however lower than expected, since IL4–10 FP highSA is highly sialylated and should therefore be protected against clearance via the ASGPR. Blockade of the ASGPR in rats injected with IL4–10 FP highSA did not affect the clearance (data not shown), suggesting other mechanisms that contribute to the initial clearance of IL4–10 FP, such as tissue distribution, or binding to other receptors, such as IL10R or IL4R on PBMCs. In addition, insufficiently capped glycoproteins can be cleared via other receptors, like the mannose receptor that binds end standing n-acetylglucosamine glycans, too. Based on our data in which the ASGPR is specifically blocked by asialofetuin, it seems that the mannose receptor has a minor role in initial clearance, if any.

Although the initial clearance of IL4–10 FP lowSA differed from that of IL4–10 FP highSA, the terminal serum half-life for both IL4–10 FP lowSA and IL4–10 FP highSA were similar. This result is likely due to the presence of a fraction of IL4–10 FP with sialylated galactose-residues in the batch of IL4–10 FP lowSA. Indeed, glycan analysis of IL4–10 FP lowSA, though predominantly showing ECL binding, revealed significant MAA binding too, pointing to the presence of galactose-residues capped with sialic acids.

Pharmacokinetics in humans of both IL-4 and IL-10 have been studied and indicated a relative short half-life for both cytokines. The apparent serum half-life of recombinant human IL-4 is 19 minutes (24), while the serum half-life of recombinant human IL-10 is much longer, 2.7 to 4.5 h (25). Indeed, we also observed short half-lives for both cytokines in rats, with IL-4 being cleared more rapidly than IL-10. Homodimerization of IL-10 increases the size of IL-10, compared to that of IL-4 (36 kDa *vs* 15 kDa). This difference in size impacts the clearance via the kidneys that is mainly affected by the size and the charge of a protein. IL4–10 FP highSA is expressed as a dimer with a molecular weight of ~70 kDa^7^. Indeed, we observed a considerably increased serum half-life of IL4–10 FP highSA (approximately 21 min), which underlines the contribution of the molecular weight to the clearance rate.

IL-4 and IL-10 are potent anti-inflammatory cytokines that in preclinical studies yielded promising results (26,27). Yet clinical results with recombinant IL-4 and IL-10 were disappointing, possibly because these cytokines have limitations when used as stand-alone therapeutic molecules. These limitations include poor pharmacokinetics due to rapid renal clearance, failure to suppress multiple pro-inflammatory mediators in inflammatory diseases in absence of other anti-inflammatory cytokines, and counterbalance of anti-inflammatory effects by their immune-stimulating activities. We postulate that a fusion protein of IL-4 and IL-10 may in part overcome the limitations of stand-alone IL-4 or IL-10 since a) the IL-4 moiety of IL4–10 FP neutralizes the immune-stimulating activities of IL-10 such as enhanced expression of activating Fc-receptors (8); b) IL4–10 FP has synergistic effects, combining the functional activities of two anti-inflammatory cytokines; and c) improved half-life of IL4–10 FP by reduced renal clearance compared to wild-type IL-10 or IL-4. Indeed, superior efficacy of IL4–10 FP compared to (a combination of) wild-type IL-4 and IL-10 was observed in inflammatory pain models (7). Here we report two different glycoforms of IL4–10 FP with similar *in vitro* potency and different clearance from the circulation. Importantly, both forms effectively reduce inflammatory pain upon local, intrathecal administration. Thus, IL4–10 FP lowSA is an attractive option for local administration as the risk for systemic effects in case of leakage from the local compartment will be minimal due to a rapid clearance by the liver. Conversely, IL4–10 FP highSA might be suitable for systemic therapy since it provides improvements compared to stand-alone IL-10 and IL-4 such as a longer serum half-life and synergistic therapeutic effects. Future studies are warranted to evaluate the clinical potential of either molecule.

## Abbreviations

ASGPR: Asialoglycoprotein receptor
BCA: Bicinchoninic acid (assay)
BSA: Bovine serum albumin
CHO: Chinese hamster ovary
CL: Clearance rate (ml/min)
CNBr: Cyanogen bromide (−activated sepharose)
ECL: Enhanced chemiluminescence (western blotting reagents)
ECL: *Erythrina cristagalli* Lectin (lectin-based ELISA)
ELISA: Enzyme linked immunosorbent assay
FP: Fusion protein
HEK: Human embryonic kidney (cells)
HRP: Horseradish peroxidase
IgG: Immunoglobulin class G antibody
IL: Interleukin
IL-1RA: IL-1 Receptor antagonist
kDa: kilodalton
LPS: Lipopolysaccharide
MAA: *Maackia amurensis* lectin II
mAb: Monoclonal antibody
nM: nanomolar
OA: Osteoarthritis
OD: Optical density
PBMC: Peripheral blood mononuclear cells
PBS(−T): Phosphate buffered saline (+ Tween-20)
PCD: Production (in picogram) per cell per day (pg/cell/day)
P/S: Penicillin-streptomycin
RA: Rheumatoid arthritis
SA: Sialic Acid
SIR: Sampling importance resampling
sTNFR: soluble TNF receptor
T_1/2_: biological half-life (min)
TNF: Tumor necrosis factor
TMB: Tetramethylbenzidine
V: Volume of distribution (ml)
